# Thyrotoxic Cardiomyopathy: State of the Art

**DOI:** 10.17925/EE.2023.19.1.78

**Published:** 2023-02-07

**Authors:** Juan Eduardo Quiroz-Aldave, María del Carmen Durand-Vásquez, Carlos Jhonatan Lobato-Jeri, Juan-Manuel Muñoz-Moreno, Diana Carolina Deutz Gómez Condori, Sofía Pilar Ildefonso-Najarro, Felipe Contreras-Yametti, Francisca Zavaleta-Gutiérrez, Luis Concepción-Urteaga, Marcio José Concepción-Zavaleta

**Affiliations:** 1. Division of Medicine, Hospital de Apoyo Chepén, Chepén, Perú; 2. Division of Family Medicine, Hospital de Apoyo Chepén, Chepén, Perú; 3. Division of Cardiology, Clínica La Luz, Lima, Perú; 4. Division of Cardiology, Hospital Nacional Edgardo Rebagliati Martins, Lima, Perú; 5. Division of Endocrinology, Hospital Nacional Guillermo Almenara Irigoyen, Lima, Perú; 6. Division of Internal Medicine, WellStar Health System Cobb Hospital, Austell, GA, USA; 7. Division of Neonatology, Hospital Belén de Trujillo, Trujillo, Perú; 8. School of Medicine, Universidad Nacional de Trujillo, Trujillo, Perú; 9. Division of Endocrinology, Clínica Javier Prado, Lima, Perú

**Keywords:** Cardiomyopathies, heart failure, hyperthyroidism, thyrotoxicosis, thyroid

## Abstract

Thyroid hormones, mainly triiodothyronine, have genomic and non-genomic effects on cardiomyocytes related to the contractile function of the heart. Thyrotoxicosis, which is the set of signs and symptoms derived from the excess of circulating thyroid hormones, leads to increased cardiac output and decreased systemic vascular resistance, increasing the volume of circulating blood and causing systolic hypertension. In addition, the shortening of the refractory period of cardiomyocytes produces sinus tachycardia and atrial fibrillation. This leads to heart failure. Approximately 1% of patients with thyrotoxicosis develop thyrotoxic cardiomyopathy, a rare but potentially fatal form of dilated cardiomyopathy. Thyrotoxic cardiomyopathy represents a diagnosis of exclusion, and prompt identification is crucial as it is a reversible cause of heart failure, and heart function can be recovered after achieving a euthyroid state using antithyroid drugs. Radioactive iodine therapy and surgery are not the best initial therapeutic approach. Moreover, it is important to manage cardiovascular symptoms, for which beta blockers are the first-line therapeutic option.

Thyrotoxicosis refers to the signs and symptoms derived from excess circulating thyroid hormones in the body,^1^ which must be differentiated from hyperthyroidism, in which there is an increase in the synthesis and secretion of hormones by the thyroid gland.^2^ Approximately 1% of patients with thyrotoxicosis develop thyrotoxic cardiomyopathy (TCM), which is a rare but potentially lethal form of dilated cardiomyopathy that causes severe impairment of left ventricular function and leads to cardiogenic shock.^3,4^ Early diagnosis is crucial as the patient is critically ill and needs urgent supportive therapy in the intensive care unit.^4^ Graves’ disease is the most common cause of hyperthyroidism and the most frequently associated with TCM.^5^ Beyond this, uncontrolled hyperthyroidism can cause cardiomyopathy.^6^ However, these structural and functional cardiac alterations are potentially reversible after achieving a euthyroid state.^3^ We describe some historical milestones related to TCM in *Figure 1*.^7–11^

The objective of this updated narrative review is to describe the pathophysiology, diagnosis and treatment of TMC in order to aid early diagnosis and treatment. A narrative review was performed, commencing with a search for articles in PubMed, MEDLINE and the Scientific Electronic Library Online databases using the Medical Subject Heading terms “cardiomyopathies” and “thyrotoxicosis”. Data published in Spanish and English during the period 2005–2022 was collated. The research was limited to articles related to humans.

Systematic reviews, clinical trials, prospective cohort studies, cross-sectional and retrospective studies, narrative reviews, case reports and case series related to the objective of this manuscript were included. We did not include letters to the editor or conference proceedings. Only studies in Spanish and English were included. A total of 91 citations were identified, of which 33 were removed based on eligibility criteria. Fifty-eight articles met the inclusion criteria and were used for the development of this narrative review.

The quality of this review was evaluated using the Scale for the Assessment of Narrative Review Articles, which includes the following items: explanation of the importance of the review, statement of objectives, description of the literature search, references, scientific reasoning and appropriate presentation of information. Twelve points were obtained corresponding to all the required items.^12^

## Pathophysiology

Thyroxine (T4) represents 85% of the hormonal production of the thyroid gland, and is converted into its active form, triiodothyronine (T3), by 5’-monodeiodinases. This occurs mainly in the liver, kidneys and skeletal muscles.^13–15^ Thyroid hormones enter and leave cells through different transporters, such as monocarboxylate transporters 8 (MCT8) and 10 (MCT10), which are present in the heart and mainly allow transportation of T3.^7,16^The myocardium does not have the enzymes to carry out deiodination, so it depends mainly on T3.^17^

**Figure 1:**
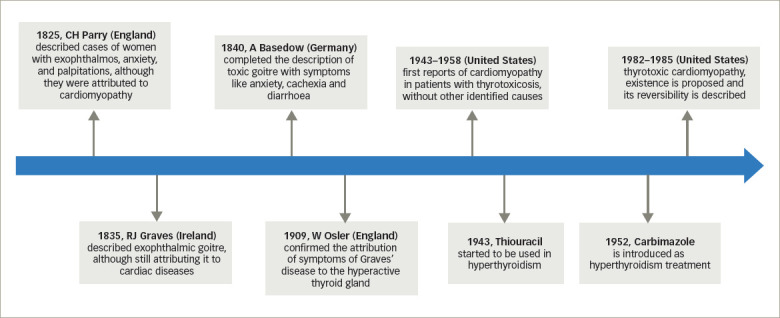
Thyrotoxic cardiomyopathy research timeline

**Figure 2:**
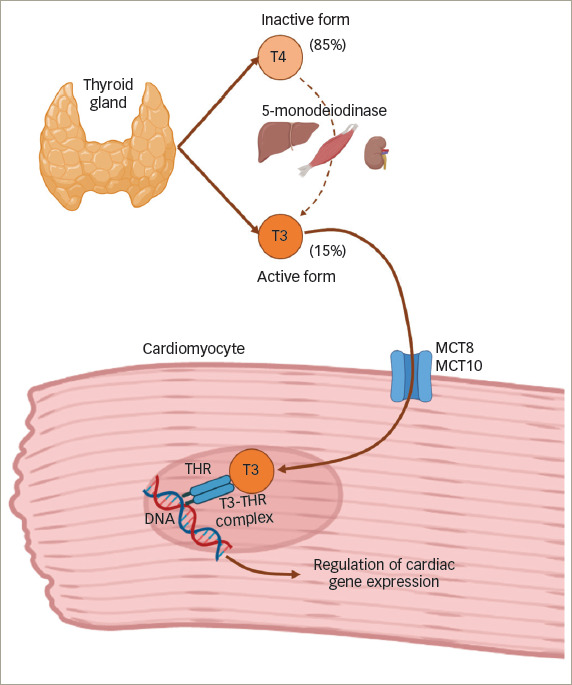
Thyroid hormone production and regulation of cardiac gene expression

The classic effects of thyroid hormone on the transcription of specific genes in cardiomyocytes are mediated by the binding of T3 to thyroid hormone receptors (THR).^3^ The T3–THR complexes translocate to the nucleus to activate or repress the expression of genes (specifically those that regulate the calcium cycle in the cardiac myocyte), thus functioning as nuclear transcription factors regulated by thyroid hormones (*Figure 2*).^7^

The binding of T3 to TRH positively regulates the expression of several cardiac genes related to the contractile function of the heart, such as α-myosin heavy chains, sarco/endoplasmic reticulum free calcium (Ca^2+^) ATPase 2 (SERCA2a), voltage-gated potassium channels (Kv1.5 and Kv4.2), Na^+^/K^+^ ATPase and β1-adrenergic receptor adenine nucleotide translocase.^7,18^ Conversely, they negatively regulate the expression of myosin β heavy chains, phospholamban (SERCA2a inhibitor), Na^+^/Ca^2+^ exchangers (NCX1), adenylate cyclase types V and VI, and THR α1.^7,18^ The contraction and relaxation of cardiac muscle are regulated by the concentration of intracellular Ca^2+^, which is determined by the release of sarcoplasmic calcium through ryanodine receptors and its reuptake by SERCA2a.^19,20^

Thyrotoxicosis markedly increases the expression of positive regulated genes and further decreases that of negatively regulated ones.^7,21^ These changes in gene expression contribute to enhanced diastolic function, as well as inotropism and chronotropism, increasing cardiac output. The non-genomic effects of thyroid hormones involve the membrane channels of sodium, potassium and calcium, and the endothelial smooth muscle and endothelial nitric oxide synthase.^7^ Together, these effects can decrease systemic vascular resistance by 50–70%,^22^ which, added to the increase in cardiac output, stimulates the juxtaglomerular apparatus, increasing the production of renin and aldosterone and sodium renal absorption, thus increasing circulating blood volume and end-diastolic volume.^17,23^ Thyroid hormones also stimulate erythropoietin synthesis, adding more circulating blood volume by up to 25%.^17^ All of this contributes to increasing preload, decreasing afterload and increasing cardiac output by up to 300%.^3^ Systolic blood pressure increases and isolated systolic hypertension can occur, mainly if arterial distensibility is reduced through atherosclerosis.^24,25^

*MCT8/10 = monocarboxylate transporter 8/10; T3 = triiodothyronine; T4 = tetraiodothyronine (thyroxine); THR = thyroid hormone receptor.*

Another effect of excess thyroid hormones is the increased rate of systolic and diastolic depolarization and the shortened refractory period of myocytes, which together produce sinus tachycardia.^17^ The shortened refractory period leads to atrial fibrillation. As there are more β-adrenergic receptors on the surface of atrial cardiac myocytes, atrial sensitivity to thyroid hormones is greater.^26^ For this reason, the frequency of atrial fibrillation and supraventricular extrasystoles is higher than in the general population, but the frequency of ventricular arrhythmias is similar. Similarly, androgens increase the expression of β-adrenergic receptor genes. As a result, susceptibility to arrhythmias is greater in men.^27^ The β-adrenergic receptor density is also altered, raising tissue sensitivity to catecholamines, even though serum catecholamines are unchanged or low in thyrotoxicosis. This leads to the increasing of positive inotropic, dromotropic and chronotropic heart rates.^28^

**Figure 3:**
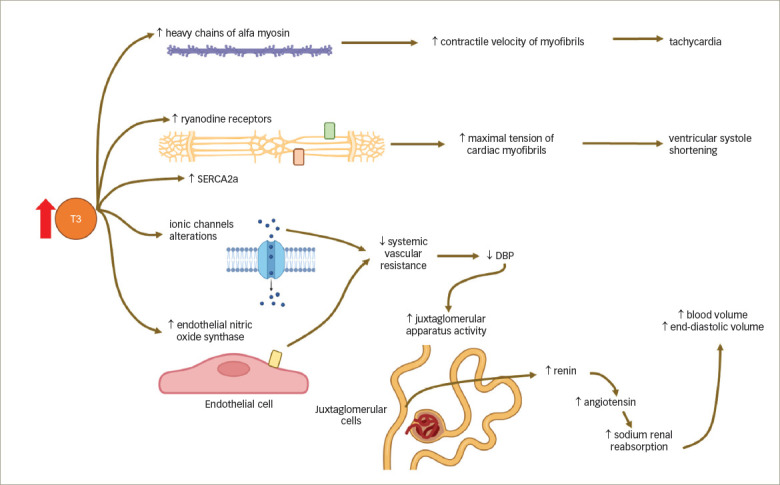
Genomic and non-genomic effects of excess thyroid hormones on the cardiovascular system

*DBP = diastolic blood pressure; SERCA2a = sarco/endoplasmic reticulum Ca*^2+^ *ATPase 2; T3 = triiodothyronine.*

Conversely, pheochromocytomas (catecholamine-hyperproducing tumours) are also capable of inducing severe cardiomyopathy (called catecholamine-induced cardiomyopathy) in a concentration-dependent manner, decreasing cardiomyocyte viability by stimulating β-adrenergic receptors, which also increases systolic blood pressure, alters left ventricular contractility, and leads to left ventricular remodelling (including hypertrophy and dilation) and dysfunction.^29–32^ Tachycardia and dysfunction of the left ventricle can lead to heart failure, being the most common pathway. In addition, volume overload causes right ventricular failure, being the second pathway of heart failure in thyrotoxicosis.^17^ Some of the genomic and non-genomic mechanisms by which excess thyroid hormones can cause cardiomyopathy are summarized in *Figure 3*.

While systemic mean arterial pressure decreases, pulmonary arterial pressure increases. This could be due to an increase in pulmonary blood flow that is not accompanied by a proportional decrease in pulmonary vascular resistance. Pulmonary arterial hypertension can result in an increased load on the right ventricle, leading to its dilation and increased right atrial pressure and central venous pressure. One consequence of this is right-sided heart failure.^33^

The preload (measured as left ventricle end-diastolic volume) and cardiac performance (measured as cardiac output) are related according to the Frank-Starling Law (left ventricular performance curves). Normally, cardiac performance increases as preload increases, but when the left ventricle contractility decreases (as in dilated cardiomyopathy), the cardiac performance for a given preload is minor.^34,35^

## Diagnosis

TCM is a diagnosis of exclusion.^23,33^ Timely diagnosis of this group of patients is important, as it constitutes a reversible cause of heart failure within 6 months of reaching a euthyroid state.^33^ The signs and symptoms of heart failure are related to a structural and/or functional alteration of the heart related to hyperthyroidism. Initially, a so-called ‘high-output’ heart failure occurs because cardiac output can increase by 50–300% as a result of the combination of increased resting heart rate, contractility, ejection fraction, systolic volume and decreased systemic vascular resistance. In advanced stages, as a consequence of poor control of thyroid function, dilated cardiomyopathy may occur with systolic dysfunction of the left ventricle and low cardiac output.^33^

### Clinic

The hyperthyroid patient usually presents with irritability, insomnia, anxiety, sweating, increased intestinal peristalsis, decreased libido, palpitations and exercise intolerance.^15,33^ On examination, patients with overt hyperthyroidism may present with hyperactivity, rapid speech, stare, lid lag, warm and moist skin, thin and fine hair, tachycardia, irregular pulse, systolic hypertension, hyperdynamic precordium, finger tremor, muscle weakness, hyperreflexia and exophthalmos. Thyroid palpation may or may not show enlargement or pain of the gland.^36^ Clinical manifestations related to high-output heart failure include general findings such as dyspnoea, tachypnoea, abdominal bloating, peripheral oedema, fatigue and signs of pleural effusion. Specific manifestations associated with thyrotoxicosis were described above.^37^

Three progressive stages have been described in heart failure due to thyrotoxicosis: the first, ‘hyperkinetic’, is characterized by preserved left ventricular systolic function that does not increase during physical exertion; the second, ‘normokinetic’, is where there is compensatory reversible myocardial hypertrophy to maintain a preserved cardiac output; and the third, ‘hypokinetic’, is a decompensated stage in which there is hypertrophy and reversible or irreversible dilation of the cardiac chambers, which translates into a decrease in the systolic function of the left ventricle and low cardiac output.^17,33^

### Laboratory

The measurement of thyroid-stimulating hormone (TSH) is the initial diagnostic test of choice. Thyroid hormones exert negative feedback on the pituitary gland so that their elevation will lead to a decrease in TSH levels. This decreased TSH is usually followed by the measurement of free T4 and total T3 levels.^38,39^ If an autoimmune process is suspected, the evaluation will continue with the measurement of serum levels of TSH-receptor antibodies,^40^ antithyroid peroxidase antibodies and thyroid-stimulating immunoglobulin.^23,33^

Previous reports described a correlation between the levels of brain natriuretic peptide (BNP) and the levels of thyroid hormones, noting that the level of BNP was four times higher in hyperthyroidism than in a euthyroid state. This is due to the fact that thyroid hormones stimulate BNP secretion by stretching the atrial myocardial tissue, and through the direct action of free T3 hormone on the increase in BNP secretion from myocardial cells due to increased gene expression.^23^

### Electrocardiography

The most frequently reported arrhythmia is sinus tachycardia (42–73%), followed by atrial fibrillation (9–23%).^26,33^ Other less frequent types include extrasystoles (5–7%), atrioventricular block (2.7–5%), atrial flutter (1.2–2.3%) and paroxysmal supraventricular tachycardia (0.2–3.3%).^26^ In some cases, patients with hyperthyroidism undergoing treatment report chest pain associated with electrocardiographic changes, such as alterations in the ST segment and the T wave, simulating myocardial ischaemia, and advanced stages of TCM with signs of volume overload can be observed.^33^ The findings described in the electrocardiogram are not specific; however, they are useful to raise suspicion of TCM, and are usually reversible after reaching a euthyroid state.^17^

### Chest X-ray

In severe thyrotoxicosis, biventricular dilatation and expansion of the pulmonary artery can be seen accompanied by right ventricular hypertrophy, simulating mitral valve disease, although without left atrial dilatation in oblique projection. Signs of pulmonary oedema can also be seen.^26,41^

### Echocardiography

Echocardiographic findings that may raise suspicion of high-output heart failure are estimated cardiac index greater or equal to 4 L/min/ m^2^, dilation of the inferior vena cava, enlargement or dysfunction of the right ventricle, estimated pulmonary artery pressure elevation, or left ventricle enlargement.^37,42^ Left ventricular hypertrophy with thickening of the posterior wall and the interventricular septum can be found.^43^ Therefore, signs of diastolic dysfunction are common, such as increased end-diastolic volume and left ventricular hypertrophy, prolonged isovolumic relaxation time (greater than 90 ms) and E-wave deceleration time (greater than 210 ms).^26^

Mitral and tricuspid regurgitation can also be identified in addition to indirect signs of pulmonary hypertension.^3^ These are caused by dysfunction of the papillary muscles. Mitral valve prolapse is a common finding, more frequently in Graves’ disease, secondary to endocardial myxomatous degeneration and glycosaminoglycan deposition, or loss of muscle tone with papillary muscle overdistention.^17^ Valvular insufficiency, in addition to the effects of thyroid hormone on pulmonary circulation, causes right ventricular failure more frequently than left ventricular failure in thyrotoxicosis.^26^ In a small percentage, dilation of all four chambers and reduced systolic function can be seen.^44,45^

### Stress echocardiography

Stress echocardiography is useful to evaluate myocardial functional reserve, which is reduced in thyrotoxicosis. This is due to tachycardia at rest and low systemic vascular resistance, which limits adaptation to exercise.^17^

### Scintigraphy

Using thallium 201 chloride, a diffuse or focal decrease in the metabolic activity of the myocardium can be evidenced.^3^

### Cardiac magnetic resonance

Cardiac magnetic resonance has a special application, primarily intended for one of the cardiac manifestations of thyrotoxicosis (autoimmune myocarditis; a form of low-output cardiomyopathy), capturing evidence of the three basic phenomena underlying myocardial inflammation (oedema, increased capillary leak and necrosis – focal fibrosis). One study found a good correlation with endomyocardial biopsy patterns.^46^

### Proposed diagnostic protocol

**Figure 4:**
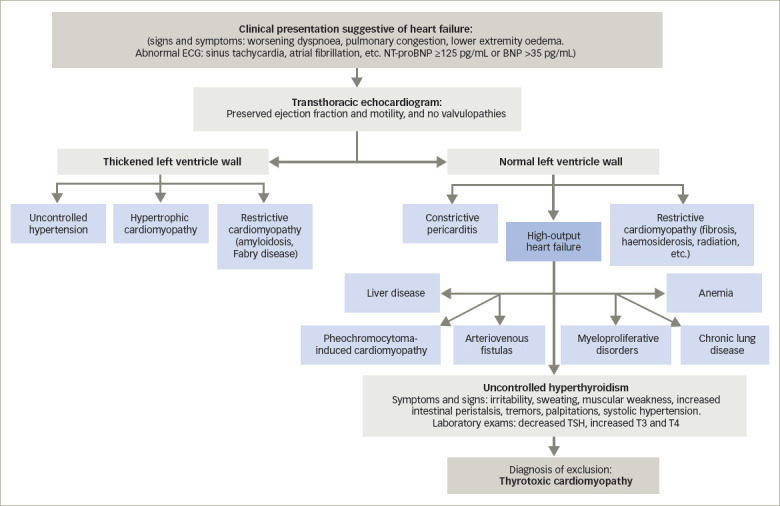
Thyrotoxic cardiomyopathy diagnostic algorithm

Currently, the basic diagnostic strategy consists of determining TSH levels and excluding other causes of structural and functional cardiac alterations, using the tools described above.^17^ With this reasoning, we propose the algorithm in *Figure 4*.

## Treatment

The goal of the treatment in patients with TCM is to restore euthyroidism and to manage cardiovascular manifestations using oral antithyroid drugs.^47^ The achievement of permanent euthyroid status is of great importance as it is associated with a better prognosis and improves cardiovascular parameters, such as heart rate, number of extrasystoles and cardiac output.^7^

Treating TCM using surgery or radioactive iodine instead of antithyroid drugs is not easy,^26^ as surgical treatment is associated with high perioperative and intraoperative risk and radioactive iodine therapy is associated with worse prognosis. The latter is due to the development of radioactive thyroiditis leading to ‘leakage’ of preformed T4 and T3, which results in recurrence of atrial fibrillation, vasospasm and cardiovascular events (myocardial infarction, stroke, pulmonary thromboembolism).^9^ Currently available drugs are methimazole and propylthiouracil, which have been used since 1940.^9^ Both drugs work by inhibiting thyroid hormone synthesis. Propylthiouracil also inhibits the peripheral hepatic conversion of T4 to T3. T3 levels have been reported to decrease 4–8 hours after administration of 200 mg of propylthiouracil, which may be useful for patients with severe thyrotoxicity such as TCM.^3^

*BNP = brain natriuretic peptide; ECG = electrocardiogram; NT-proBNP = N-terminal pro-BNP; T3 = triiodothyronine; T4 = tetraiodothyronine (thyroxine); TSH = thyroid-stimulating hormone.*

In Graves’ disease, a regimen starting with high doses of antithyroid medications (30–60 mg/day of methimazole or 300–600 mg/day of propylthiouracil) is usually prescribed and may be titrated at intervals of 4–8 weeks according to clinical response and serum thyroid hormone levels, evaluating therapeutic efficacy and guiding the dose to achieve euthyroidism.^39^ The American Thyroid Association recommends using initial doses of methimazole based on free T4 levels, as shown in *Table 1*.^39^ Treatment should be administered for 6 months to 2 years, although the general recommendation is 18 months of continuous treatment.^39^ It is important to consider the side effects of antithyroid drugs, such as rash in up to 5% of patients and agranulocytosis in 0.5%. Other less frequent side effects include cholestatic jaundice, hepatocellular toxicity, angioneurotic oedema and arthralgias.^17^ The overall remission rate with treatment is in the range of 50% and can be as high as 80% in subgroups with mild disease.^39,48^ There is some evidence that continuation of treatment with antithyroid drugs beyond what is usually recommended may be associated with higher remission rates.^39,49^ Radioactive iodine therapy is usually recommended for older adults. However, long-term administration of an antithyroid drug is safe and some patients may prefer it rather than a destructive therapy. This is one of the current recommendations of the European Thyroid Association.^49^

The management of congestive heart failure with impaired systolic function usually includes fluid and sodium restriction, and administration of diuretics, vasodilators, renin–angiotensin system antagonists and beta blockers.^48,50^ Beta blockers are safe and effective drugs for relieving symptoms, being the first choice for reducing heart rate in patients with hyperthyroidism.^39^

**Table 1:** Methimazole dosage based on elevation of free thyroxine above the upper limit of normal

Free T4 (times above the normal level if the limit is 2 ng/dL) (measurement)	Dose of methimazole (mg/day)
1.0–1.5 (2–3 ng/dL)	5–10
1.5–2.0 (3–4 ng/dL)	10–20
2.0–3.0 (4–6 ng/dL)	30–40

*Adapted from Ross DS et al., 2016*.^39^

Treatment with propranolol (loading dose: 0.15 mg/kg intravenous [IV]; maintenance dose: 80–240 mg daily in divided doses), atenolol (25–100 mg, once or twice daily), metoprolol (loading dose: 2.5–5 mg IV bolus over 2 minutes; maintenance dose: 25–100 mg twice daily), or other beta blockers is recommended until thyroid hormone levels have normalized. The goal is to decrease heart rate rapidly, relieve left ventricular dysfunction related to the ventricular rate in hyperthyroidism, prevent palpitations, lower systolic blood pressure and reduce muscle weakness and exercise intolerance.^17,38,51^

Beta blockers should be considered in all patients with TCM and other patients with thyrotoxicity with resting heart rates greater than or equal to 90 beats per minute or with coexisting cardiovascular disease. In patients with asthma, beta blockers should be avoided because of concerns regarding bronchoconstriction.^52^ Cardioselective beta blockers are better tolerated, but not completely safe. The risk of bronchospasm can be decreased by using the lowest possible dose and a beta-blocker with higher β1 selectivity.^53^ The use of beta blockers in asthma and TCM could be based on an individual risk assessment.^54^

**Figure 5:**
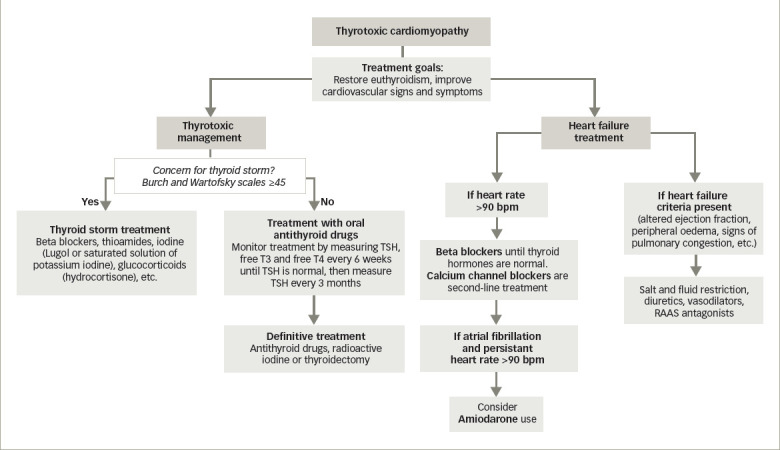
Thyrotoxic cardiomyopathy therapeutic algorithm

*bpm = beats per minute; RAAS = renin–angiotensin–aldosterone system; T3 = triiodothyronine; T4 = tetraiodothyronine (thyroxine); TSH = thyroid-stimulating hormone.*

The mainstay of atrial fibrillation management in patients with hyperthyroidism is beta blockers and an antithyroid agent (propylthiouracil or methimazole).^55,56^ In general, digoxin is not recommended to treat atrial fibrillation in hyperthyroidism because it increases renal clearance and sympathetic tone and decreases vagal tone. In addition, it requires a large volume of distribution, needing a higher dose of digoxin than usual, which can result in digoxin toxicity.^55^

For patients in whom the use of beta blockers is contraindicated, the careful use of calcium channel blockers like verapamil (loading dose: 0.075–0.15 mg/kg IV over 2 minutes; maintenance dose: 120–360 mg daily in divided doses) and diltiazem (loading dose: 0.25 mg/kg IV over 2 minutes; maintenance dose: 5–15 mg per hour IV infusion or 120–360 mg daily in divided doses) could be recommended for heart rate control, as they enhance the reduction of blood pressure through their effects on smooth muscle cells of the arterioles. Consideration needs to be given to cardiovascular shock and hypotension in the use of these drugs.^17,51^ Digitalis and diuretics may be administered to patients with severe heart failure and pulmonary oedema. Both drugs are also safe and effective when given together with beta blockers.^55^

Calcium channel blockers should be avoided in reduced ejection fraction or haemodynamic instability due to their strong negative inotropic effect. Amiodarone can be used in these acute settings because it allows the patient to return to a sinus rhythm when combined with antithyroid medications and reduces the possibility of worsening thyrotoxicosis. The recommended loading dose is 800 mg daily for 1 week, followed by 600 mg daily for 1 week, then 400 mg daily for 4–6 weeks; the maintenance dose is 200 mg daily.^51,56^

Nearly two-thirds of patients return to normal sinus rhythm 8–10 weeks after achieving a euthyroid state.^57^ In those patients with persistent atrial fibrillation despite achieving a euthyroid state, rhythm control may be an option. However, as in the general population with atrial fibrillation, rate control is generally initially preferred. Therapeutic options include class IA, IC and III antiarrhythmics.^56^

The use of amiodarone may be indicated acutely, as mentioned above, during a thyroid storm to restore sinus rhythm or as chronic treatment in patients with atrial fibrillation refractory to rate control.^56^

The use of anticoagulation in patients with hyperthyroidism and atrial fibrillation has been controversial, although hyperthyroidism has been shown to increase the risk of thrombotic events in patients with atrial fibrillation regardless of CHADS2-VASc score.^56,58^ However, the optimal use of anticoagulants represents a therapeutic dilemma due to the increased risk of bleeding. Thyroid disorders affect haemostatic balance, thus affecting the efficacy of anticoagulants in patients with atrial fibrillation.^58^ The risk of systemic or cerebral embolization must be weighed against the risk of bleeding and other complications of this therapy.^51,56,58^ The treatment regimen is summarized in *Figure 5*.

## Conclusions

TCM is a rare but potentially lethal complication of thyrotoxicosis, which is reversible if euthyroid status is achieved. As it is a diagnosis of exclusion, additional tests are of great importance in the diagnostic approach. Antithyroid drugs are the first-line treatment.

### Limitations

All authors recognize that, despite describing the methodology used for the development of this narrative review, a research question is not answered; therefore, the scientific evidence is less than a systematic review. Additionally, a minor limitation is the absence of clinical practice guidelines relating to the diagnosis and management of TCM.
